# Mechanochemically Activated Aluminosilicate Clay Soils and their Application for Defluoridation and Pathogen Removal from Groundwater

**DOI:** 10.3390/ijerph16040654

**Published:** 2019-02-22

**Authors:** Olumuyiwa A. Obijole, Mugera W. Gitari, Patrick G. Ndungu, Amidou Samie

**Affiliations:** 1 Environmental Remediation and Nanoscience Research group (EnviReN), Department of Ecology and Resource Management, School of Environmental Sciences, University of Venda, Private Bag X5050, Thohoyandou 0950, South Africa; obijoleoa@aceondo.edu.ng; 2 Department of Applied Chemistry, Faculty of Science, University of Johannesburg, Post Office Box 524, Auckland Park, Johannesburg 2006, South Africa; pndungu@uj.ac.za; 3 Department of Microbiology, School of Mathematical and Natural Sciences, University of Venda, Private Bag X5050, Thohoyandou 0950, South Africa; amidou.samie@univen.ac.za

**Keywords:** adsorption capacity, bacteria, defluoridation, *Escherichia coli*, clay soil, fluoride, mechanochemical activation, pathogen

## Abstract

In this study, aluminosilicate rich clay soils were prepared through mechanochemical activation. The chemical and mineralogical properties were investigated using X-Ray Fluorescence (XRF) and X-ray diffraction (XRD). The functional groups, morphology and surface area were evaluated using Fourier Transform Infra-Red (FTIR), Scanning electron microscopy (SEM) and Brunauer-Emmett-Teller (BET) analysis. Batch experiments were used to evaluate its defluoridation efficiency while antibacterial activities were assessed using well diffusion method. Maximum adsorption capacity was found to be 1.87 mg/g with 32% fluoride removal. Fluoride adsorption was found to reduce in the presence of Cl^−^, PO_4_^2−^ and CO_3_^2−^ while it increased in the presence of SO_4_^2−^ and NO_3_^−^. Adsorption data fitted well to Freundlich isotherms, hence, confirming heterogeneous multilayer adsorption. Kinetic studies revealed that fluoride adsorption fitted well to pseudo-second order model. The sorption of F^−^ onto the clays’ surface followed intra-particle diffusion mode. High correlation coefficient indicates that the sorption process was greatly controlled by particle diffusion while it is minimal in pore diffusion model. Antibacterial studies revealed no zone of inhibition for all the activated clays, hence indicating that they are not active against the bacterial strains of *Escherichia coli* used in this study. The results showed activated clays’ potential for defluoridation. Its effectiveness in pathogen removal is limited. Hence further modifications of the clays’ surfaces are hereby recommended.

## 1. Introduction

Water is an essential resource necessary for human health and survival. Groundwater is one of the major sources of potable water which is becoming increasingly contaminated as a result of natural and anthropogenic activities worldwide. Fluoride is one of the various water contaminants. High fluoride levels above the permissible limit cause fluorosis. The presence of pathogens in water leads to various types of water borne diseases. Different types of clays and some metal oxide-coated aluminosilicate materials have been used in defluoridation and pathogen removal, but they are not totally active against bacterial strains. Clay minerals are naturally occurring fine grained phyllosilicate minerals which impart plasticity to clay and harden when dried or fired [1]. Our knowledge about the structure of clay minerals has significantly increased over the years. Its use has widened greatly due to advances in instrumental and analytical methods such as x-ray diffraction, electron microscopy and spectroscopy. Attention is now focusing on clay properties as natural nano-sized particles which are applied in adsorption, catalysis, and in nanotechnology research on synthetic and natural materials for the removal of contaminants from water [1,2]. Clays generally contain the following main elements: aluminum, silicon and oxygen, others are iron, magnesium, alkali metals, alkaline earths, and other cations present either in the interlayer space or in the lattice framework by isomorphous substitution [3]. Clay deposits are broadly classified into three classes according to the dominant clay minerals present. These include: (a) Kaoline fields where the dominant clay mineral is kaolinite. (b) Bentonite fields in which the dominant clay mineral is montmorillonite. This forms part of the smectite group. (c) Palyorskite fields with palygorskite as dominant clay mineral [1]. Adsorption studies of fluoride on clays and bauxite show that bauxite has the best adsorption capacities, followed by bentonite and palygorskite, while kaolinite had the lowest adsorption capacity [2]. Other adsorbents studied for defluoridation are laterite [4], mechanochemically activated kaolinites [5], China clay [6], bentonite and montmorillonite [7]. Various studies have been carried out by subjecting clay to high temperatures to produced clay wares with optimal fluoride binding capacity. The results of these studies showed that clay could be a promising choice adsorbent for defluoridation of water [8]. Studies have been done on the use of red mud, coal fly ash and clay for defluoridation [9,10,11,12]. Some metal oxide-coated adsorbents obtained from aluminosilicate materials are good at defluoridation, but not totally active against bacterial strains [13,14,15]. The main objective of this work is to determine the physico-chemical and mineralogical characteristics of the clay soil from Mukondeni Village, Limpopo, South Africa, with a view to ascertaining its suitability as adsorbents for simultaneous defluoridation and pathogen removal from groundwater. This was done by investigating: i) the geological fluoride present in the activated clay, point of zero charge and CEC; ii) the chemical and mineralogical properties using XRF and XRD; iii) The functional groups, morphology and the surface area using FTIR, SEM-EDS and BET; iv) the activated clays adsorptive capacities by batch mode; v) the effect of co-existing ions on the fluoride uptake; vi) adsorption isotherms modelling from data generated; vii) the adsorption kinetics modelling from generated data; viii) the intra-particle modelling from data generated; ix) antibacterial activities using the well diffusion method.

## 2. Materials and Methods

### 2.1. Sample Collection and Preparation

Clay soil samples used were sourced from the village of Mukondeni in Vhembe District, Limpopo, South Africa. The clay materials were separated from foreign visible coarse particles manually, cleaned, air-dried followed by oven drying to constant weight, ground with agate mortar and made to pass a 250 µm sieve. The finely ground dried clay powder was then mechanochemically activated at different times, labelled and kept in zip lock bags until use.

### 2.2. Preparation of Mechanochemically Activated Clay Samples

To prepare activated clay, the optimum milling time was investigated by milling a known mass of clay at different times of between 5 to 60 min at 700 rpm using a RS200 milling machine (Retsch, Green Bay, WI, USA). The obtained clay powder of <250 µm particle size was then oven dried and stored in zip lock bags. The optimum milling time was obtained by evaluation of the activated clays (samples A–F) for defluoridation at a specific pH using batch mode. The treatment above is a mechanical treatment but in the course of the milling process, some of the bonds in Al-O-Al and Si-O-Si bonds in the aluminosilicate clay matrix may have been broken. This is in fact a form of chemical reaction; hence the process is called mechanochemical treatment. Furthermore, the geological fluoride level in the activated clay were determined by using batch mode and the percent fluoride removal evaluated. This was evaluated to determine the safe threshold of geological fluoride in the clay materials in order to establish the suitability of the activated clay materials for defluoridation in groundwater.

### 2.3. Physicochemical and Mineralogical Characterization

Surface area, specific surface area pore sizes and volumes was evaluated by using the Brauner-Emmett-Teller (BET) method. The measurements were carried out using a TriStar II Surface Area and Porosity Units instrument (Micromeritics, Norcross, GA, USA). Mineralogical composition of the activated clay was analyzed using a X’Pert Pro powder diffractometer (PANanalytical, Almelo, The Netherlands) in θ–θ configuration with an X’Celerator detector and variable divergence- and fixed receiving slits with Fe filtered Co-Kα radiation (λ = 1.789Å). The phases were identified using X’Pert Highscore plus software. The relative phase amounts (weight percent) were estimated using the Rietveld method (Autoquan Program). Elemental composition was evaluated by using a PANanalytical Axios X-ray fluorescence spectrometer equipped with a 4 kW Rh tube. The activated clays’ morphology (size and shape at the surface) were investigated by sprinkling a small amount of the sample onto a scanning electron microscope (SEM) stub covered with carbon glue. The stubs were then coated with carbon in an evaporation coater. The SEM is a NanoSEM 230 (FEI Nova, Czechoslovakia Republic) with a field emission gun (FEG). The elemental analysis was carried out using an X-Max detector (Oxford, Abingdon, UK) equipped with Inca software, at 20 kV. The Fourier transform infrared (FT-IR) analysis of the activated clay, which provides information about the functional groups responsible for fluoride sorption from the groundwater, was carried out using an ALPHA FT-IR spectrophotometer (Bruker, Germany). The point zero charge ($pH_{pzc}$) was determined using titration at 0.1 M, 0.01 M and 0.001 M KCl concentrations [16]. The concentration of exchangeable cations was determined using flame atomic absorption spectra (600 PerkinElmer, Waltham, MA, USA). The Cation Exchange Capacity (CEC) was evaluated by using ammonium acetate buffers at pH 5.4 and 7.4, respectively [17].

### 2.4. Defluoridation Experiments-Optimization of Fluoride Adsorption Conditions

Batch adsorption experiments were used to assess the activated clays’ capacity for defluoridation of fluoride rich simulated water: The following parameters were optimized: contact time; adsorbent dosage; pH and fluoride ions concentrations. The effect of co-existing ions on fluoride uptake onto the clay surfaces’ were also evaluated. Modelling of the adsorption isotherm was done from the data generated using Langmuir and Freundlich equations. Adsorption kinetics studies were also carried out from the generated data. The sorption mechanism of fluoride uptake was investigated by using the pseudo-first order and pseudo-second order kinetic models. Studies on the intra-particle diffusion model was also carried out, using the data generated to determine if the sorption process during defluoridation is being controlled either by a particle diffusion or a pore diffusion model.

### 2.5. Bench Scale Pilot Testing of Fluoride Rich Siloam Groundwater with the Activated Clay

The efficacy of the activated clays for defluoridation was tested with fluoride rich groundwater collected from boreholes in Siloam village, South Africa, at the already established optimum conditions of contact time, pH, and adsorbent dosage. Adsorption of fluoride rich Siloam groundwater by the activated clay was carried out at optimum pH 6.0 and natural pH of about 8. A mixture of the pilot water and 2.0 g/100mL of activated clay with initial pH 6.0 was equilibrated for 60 min at the shaking speed of 250 rpm followed by centrifuging. The mixtures were filtered using a 0.45-µm pore membrane filter and the analysis of fluoride in the supernatant were carried out. The percent fluoride removal and adsorption capacity were evaluated. The cations and anions in the groundwater before and after treatment were analysed using Ion Chromatography on a 432 conductivity detector (Waters, Switzerland) coupled to a Waters 717plus Auto sampler and an 1100 series binary pump (Agilent, Switzerland). The cations evaluated are sodium, potassium, calcium and magnesium while the anions are bromide, chloride, nitrate, phosphate and sulphate.

### 2.6. Effect of co-Existing Anions on Fluoride Adsorption

Generally, groundwaters, including fluoride rich ones, are most likely to contain several other anions which may compete with fluoride ions uptake in the adsorption process. The effect of co-existing anions on fluoride sorption onto the clays surfaces was evaluated in the presence of 10 mg/L salt solution of chloride, sulphate, nitrate and phosphate, independently, at an initial fluoride concentration of 10 mg/L. All the solutions for fluoride adsorption experiments and analysis was prepared by an appropriate dilution from the stock solution prepared as described earlier in Section 2.4. Adsorption studies was carried out for each of the prepared 10 mg/L salt solution of chloride, sulphate, nitrate and phosphate, separately, at an initial fluoride concentration of 10 mg/L in a conical flask by batch method at room temperature. The pH of the fluoride solution was adjusted to 6.9 ± 0.1, using 0.1 M NaOH and HCl solutions. 2 g of the activated clay was introduced into each of the 100 mL of the blank, chloride, sulphate, nitrate and phosphate and placed in a shaker at a speed of 250 rpm to give homogenous mixture. The solid mixture was decanted from the mixture and the resulting solution (filtrate) after centrifuging for about 30 min. The amount of fluoride in the filtrate was analysed using a pH meter equipped with ion-selective fluoride electrode. The analysis was carried out by using total ionic strength adjustment buffer TISAB III/ Sample Solution ratio of 1:10. A blank experiment was also performed using deionized water with only 10 mg/L fluoride concentration. The percent fluoride removal was then calculated from the result obtained, and comparison made.

### 2.7. Regeneration Experiments

Regeneration of the fluoride-loaded activated clay was carried by agitating 2.0 g of loaded clay with 100 mL of 0.1 M NaOH for 30 min on a mechanical shaker. After agitation, the mixtures were filtered through a 0.45-µm pore membrane filter and the filtrate was diluted to 100 mL and then analysed for desorbed fluoride. The resulting clay adsorbent on 0.45-µm pore membrane filter paper was washed with de-ionized water and then dried in the oven at 110 °C for 4 h. Adsorption studies on the regenerated clay adsorbent was then carried out using batch method. The regenerated clay adsorbent was re-used for defluoridation up to five times.

### 2.8. Antibacterial Studies

Antibacterial activities of the six mechanochemically activated clays were evaluated with *Escherichia coli* (*E. coli*) strains by using well diffusion assay method. The zone of inhibitions was recorded in millimeter (mm). The bacterial suspensions were prepared with the turbidity of 0.5 McFarland. Mueller-Hinton agar plates were inoculated with *E. coli* (ATCC 35218) strains. Wells with a diameter of 6 mm were cut using a cork borer and filled with 30 µL of the six mechanochemically activated clay soils samples, (A-F). Plates were incubated for 24 h at 37 °C. After incubation, the growth inhibition zone diameters were measured in millimeter (mm). The plates as well as the samples were carefully packed in a sealed disposable plastic bags for total destruction immediately after the measurements were made.

## 3. Results and Discussion

### 3.1. Geological Fluoride Levels in the Mechanochemically Activated Clays

The geological fluoride levels in the activated clays at different contact times of 5 to 60 min ranged between 0.0401–0.0638 mg/L (Table 1). The fluoride levels in mg/g of the clay was also calculated and found to range between 2.0 × 10^−3^ and 3.51 × 10^−3^ mg/g (Table 1). The fluoride levels in the activated clay was found to be much lower than the World Health Organization [18,19,20] and South African National Standards permissible limits [21], whose recommended maximum fluoride limit is 1.5 mg/L. Hence, from Table 1, the geological fluoride levels in the activated clay is within safe threshold, therefore making the clay materials a safe and promising adsorbent for fluoride removal without compromising the water safety during defluoridation processes.

### 3.2. Physicochemical and Mineralogical Characterization

#### 3.2.1. Surface Area by the Brunauer-Emmett-Teller (BET) Method

The surface area, pore volume and pore size play a significant role in the sorption of the fluoride ions onto the activated clay surface. The results of a Brunauer-Emmett-Teller (BET) analysis of the activated clays at different times are presented in Table 2. They show that the clay is mesoporous in nature, with pore sizes ranging between 5–15 nm. The surface area ranged between (13–18 m^2^/g). Specific surface area ranged between (12–17 m^2^/g) while pore volume values gave an average value of 10 cm^3^/g. Clay sample D had the largest surface area (17.19 m^2^/g), specific surface area (16.66 m^2^/g), pore volume of 10.07 cm^3^ and pore size of 14.93 nm at 30 min treatment time, indicating maximum roughness of pore walls and increase of additional active sites for the activated clay while sample F has the smallest surface area (13.23 m^2^/g), specific surface area (12.58 m^2^/g), a low pore volume (10.05 cm^3^/g) and pore size of 14.68 nm respectively, at 60 min treatment time. Since surface area generally plays a significant role in the adsorption of sorbents from the solution, the higher the surface area, the higher the adsorption and vice versa [22]. The results also show that activation increases the surface area of the clay from 10 min to 30 min (17.1 m^2^/g) and thereafter decrease to about 13 m^2^/g at 60 min treatment time, Hence clay sample D which was activated for 30 min is considered optimum for the modification process since the sample has the highest surface area and pore volume suggesting that it has more active sites for fluoride uptake, thereby improving the quality of the clay as a good adsorbent for fluoride removal.

#### 3.2.2. Cation Exchange Capacity (CEC)

Determination of the cation exchange capacity (CEC) of the most activated clay was carried out using standard laboratory methods [16]. The CEC also reveals that Mg^2+^, Ca^2+^, Na^+^ and K^+^ are the exchangeable cations present. The higher the concentrations of these cationic species on the clays surfaces, the higher their adsorptive properties. CEC values of the activated clay soil at pH 7.4 and 5.4 (milliequivalent/100 g) was calculated to be 74.5 and 82.1, respectively (Table 3). This is further corroborated by XRF results. From results obtained, it can be seen that the CEC is not dependent on pH because there was not much difference in the concentration of cations at different pH. The results correlate with that reported by Mudzielwana et al. [23] and Gitari et al. [10] who concluded that CEC is independent of pH. Furthermore, it can also be concluded that the CEC of the clay soil is generally moderate compared to the smectite [23] and montromonilite [22,23].

#### 3.2.3. Point of Zero Charge of Activated Clay

pH_pzc_ of the activated clay soil evaluated was found to be 6.1 (adsorbent dosage: 1.0 g/50 mL, contact time: 24 h, agitation speed: 250 rpm and 298 K). This value is close to that of smectite rich ($pH_{pzc}$ 6.0) clay reported in [23] and lower than that of a bentonite clay soil (${\mathrm{pH}}_{\mathrm{pzc}}$ 8.2) reported in [10]. ${\mathrm{pH}}_{\mathrm{pzc}}$ refers to the pH at which the clay has zero net charges on the surface. Above the ${\mathrm{pH}}_{\mathrm{pzc}}$ the clay is negatively charged and below the ${\mathrm{pH}}_{\mathrm{pzc}}$ the clay is positively charged. Therefore, the implication of the clays’ $pH_{pzc}$ 6.1, is that adsorption of fluoride anions will most likely be higher at pH below 6.1 due to the clay surface being entirely occupied by positive charges while adsorption of fluoride anions tends to decrease at pH above this value due to the surfaces being occupied more by negative charges which tends to discourage fluoride adsorption onto the clays surfaces since like charges repel each other. Furthermore, the close ${\mathrm{pH}}_{\mathrm{pzc}}$ values of (${\mathrm{pH}}_{\mathrm{pzc}}$ 6.1) in this study and that of [23] which is (${\mathrm{pH}}_{\mathrm{pzc}}$ 6.0) could be attributable to both clays being smectite rich.

#### 3.2.4. Morphology of the Mechanochemically Activated Clay

**Figure 1:**
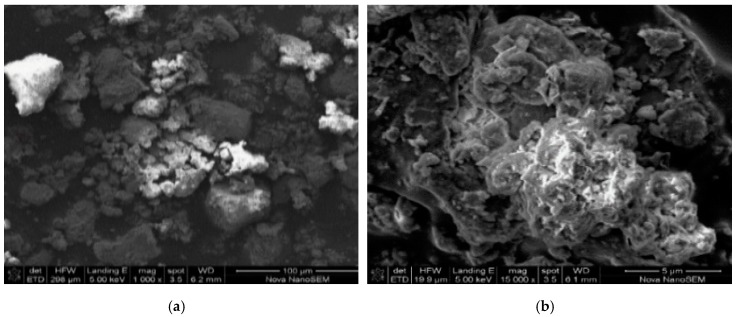

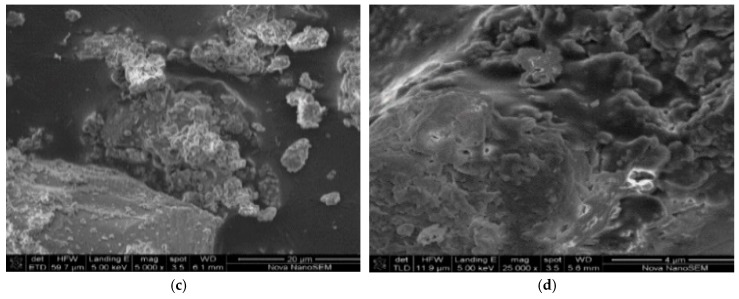
(**a**–**d**): Scanning electron microscopy (SEM) micrographs of the most mechanochemically activated clay at different magnifications.

Scanning electron microscopy (SEM, Figure 1a–d) shows the surface structure of the most activated clay. The images consist of the fine particles of irregular shape and size on external surface with a micro-rough texture. The morphology of the clay in Figure 1a and b shows an irregular porous structure at lower magnifications. At higher magnifications, the clay soils images show a smooth irregular surfaces, and an expanded honeycomb, flared, “cornflake” and platy like texture (Figure 1c,d). The characteristic images show typical smectite clay surfaces which is consistent with the results obtained by [23].

**Figure 2:**
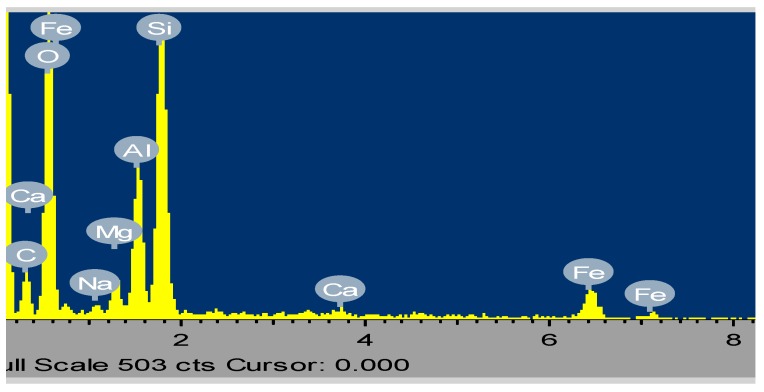
Energy dispersal spectroscopy (EDS) of the activated clay sample.

Energy Dispersal Spectroscopy (EDS) of the most activated clay is presented in Figure 2. The elemental concentrations are shown with silicon, iron, aluminium and oxygen having most elevated concentrations indicating that the clay material is an aluminosilicate material. There is also the presence of base cations of magnesium, sodium, potassium and calcium in the sampled powdered clay, which are the exchangeable fractions and plays a vital role in sorption of the fluoride onto the clay surfaces. The obtained EDS results is corroborated the XRF and XRD results.

#### 3.2.5. X-Ray Diffraction (XRD)

**Figure 3:**
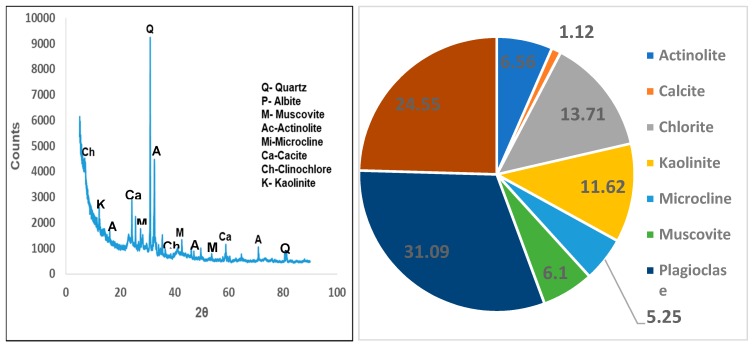
(**a**) X-ray diffraction spectrum (**b**) X-ray diffraction quantitative results of the activated clay.

X-ray diffraction (XRD) analysis shows that the activated clay sample is mainly composed of the following mineral phase which are plagioclase, quartz, chlorite, kaolinite, actinolite, muscovite, microcline, calcite and albite (Figure 3a). The quantitative results further confirm the presence of plagioclase (smectite rich clay soil) (31.09%) and quartz (24.55%) as the major minerals and the presence of chlorite (13.71%) and kaolinite (11.62%) as minor minerals (Figure 3b).

#### 3.2.6. X-Ray Fluorescence (XRF)

Table 4 presents the major and minor elemental composition of the activated clay. The XRF analysis of the activated clay shows that silica (SiO_2_) is the main component at 52.48%, followed by Al_2_O_3_ at 14.62%. High concentrations of SiO_2_ and Al_2_O_3_ reveal that they are aluminosilicate rich material. Minor elemental compositions are P_2_O_5_, TiO_2_, Na_2_O, MnO, K_2_O, CaO, MgO and Fe_2_O_3_ with their compositions ranging between 0.03 to 6.64%. Some of these minor elemental compositions form the exchangeable cations which play a vital role in fluoride uptake during defluoridation.

#### 3.2.7. Fourier Transform Infra-Red (FTIR) Analysis

**Figure 4:**
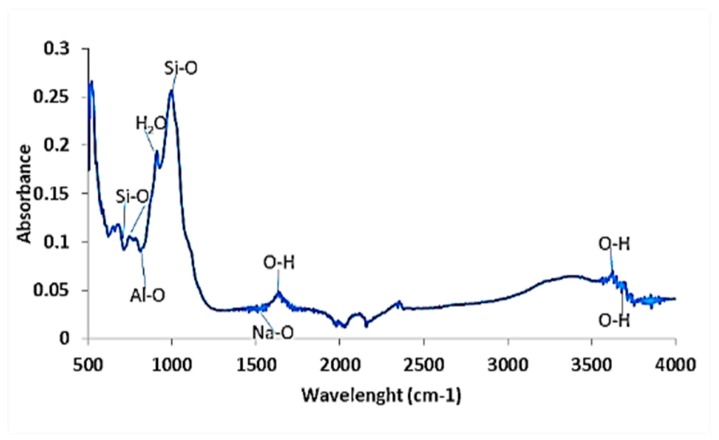
FT-IR Spectra of the activated clay sample.

The activated clay samples were scanned in the range between 4000 and 500 cm^−1^ and the spectra obtained are shown in Figure 4. The spectra show three main absorption regions: 3000–3800 cm^−1^, 1300–1800 cm^−1^ and 500–1200 cm^−1^. The hydroxyl stretching absorption bands are well established at around 3700 cm^−1^. The absorption bands observed at 3400–3500 cm^−1^ and 1600–2700 cm^−1^ could be due to the OH vibrational mode of the hydroxyl molecule, which is observed in almost all the natural hydrous silicates, including montmorillonite clay [24]. The bands between 3400 and 3700 cm^−1^ are attributed to the OH stretching mode. The H-O-H bending of water is observed at 1620−1640 cm^−1^. In the 1000 cm^−1^–500 cm^−1^region, the main functional groups were Si-O-Si and Al-O-H. The IR peak at 970 cm^−1^ may be attributed to Al-OH-Al. The presence of these functional groups and their stretching bands, particularly H-O-H, Al-O-Al and Si-O-Si, have been known to play a vital role in fluoride sorption onto the clay surfaces. This is mainly through fluoride interaction with the OH groups and direct interactions with metal surface. The IR spectra also showed Si-O stretching between 780 cm^−1^–690 cm^−1^ and around 465 cm^−1^ which is indicative of quartz presence in the clay.

### 3.3. Defluoridation of Simulated Fluoride Water with the Activated Clays

The percent fluoride removal of clays activated at different times of between 5 and 60 min is presented in Table 5. Sample D exhibited highest fluoride removal of 28% and adsorption capacity of 1.195 mg/g respectively at 30 min activation time, hence indicating optimum activation time of 30 min (Table 5). The percent fluoride removal of the activated clay was calculated as follows:

(1) $$\mathrm{Percent}\;\mathrm{Fluoride}\;\mathrm{removal}=\frac{\left(C_{0}-C_{e}\right)}{C_{0}}\times 100$$

where *C*_0_ and *C_e_* are the initial and equilibrium concentrations of fluoride solution in mg/L, respectively.

The adsorption capacity, *q* (mg/g) of the activated clay is calculated according to the equation below:

(2) $$\mathrm{The}\;\mathrm{adsorption}\;\mathrm{capacity},\;q=(C_{0}-C_{e})\times \frac{v}{m}$$

where *q* is the mass of fluoride adsorbed in mg/g of adsorbent, $C_{0}\;\mathrm{and}\;C_{e}$ are the initial and equilibrium concentrations of fluoride respectively, *v* is the volume of the solution in litres and m is the mass in gram of the clay adsorbent.

**Figure 5:**
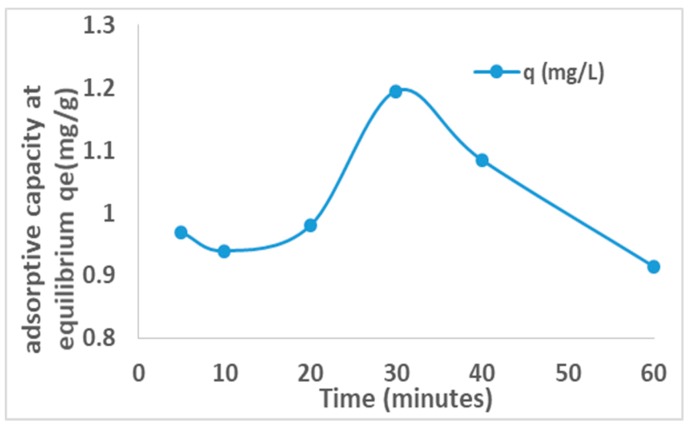
Plot of adsorptive capacity (mg/g) versus activation time (min).

The plot of adsorption capacity versus activated time is presented in Figure 5 below.

The adsorption capacities increase from 0.94 mg/g to 1.195 mg/g at 30 min activation time and reduce thereafter to 0.915 mg/g at 60 min activation, hence indicating that the activation had effect on the adsorption capacity of the clay soils. This was probably due to increase in the surface area which peaked at 30 min activation. The observed decrease in adsorption capacity after 30 min could be attributed to further reduction in pore sizes and volumes as the particle sizes decreases.

### 3.4. Optimization of Fluoride Adsorption Conditions

#### a) Effect of Contact Time

**Figure 6:**
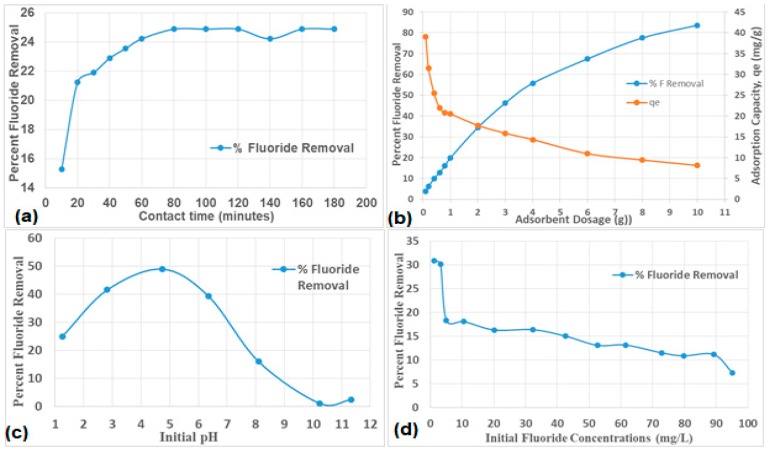
Optimisation of fluoride removal by activated aluminosilicate rich clay soil: (**a**) effect of contact time, (**b**) effect of adsorbent dosage, (**c**) effect of initial pH and (**d**) effect of initial concentration.

An increase in the percent fluoride removal with an increase in contact time was observed (Figure 6a). The rate of fluoride removal was very rapid within first 20 min of contact and thereafter stabilized at ≈ 60 min (24.5 % fluoride removal). After 60 min, the fluoride removal rate stabilized, suggesting that the system had reached equilibrium (Figure 6a). Therefore, 60 min were taken as the optimum contact time and were used for subsequent experiments.

#### b) Effect of Adsorbent Dosage

The percent fluoride removal was observed to increase with an increase in the activated clay dosage (Figure 6b). As dosage increases, more sites and surfaces become available for fluoride uptake. From 0.1 to 2.1g dosage, the fluoride uptake was observed to increase gradually to about 38% fluoride removal. At low adsorbent dosage, there is rapid fluoride adsorption since the active sites are more readily available while at high adsorbent dosage, the adsorbate species increasingly find it difficult to access the adsorption sites due to less available sites as a result of the fluoride filling of these sites. Hence gradual stabilization in adsorption process. Therefore, it can be concluded that the optimum dosage of the activated clay for defluoridation is 2.0 g/100 mL, hence it was adopted for subsequent experiments.

#### c) Effect of pH

The pH of the medium plays a significant influence on fluoride adsorption and help in understanding fluoride sorption mechanism of the adsorbent. The effect of initial pH on percent fluoride removal was evaluated by varying the initial pH of the medium from 2 to 12 by using 0.1 M HCl and 0.1 M NaOH. From the results (Figure 6c), the activated clay showed fluoride sorption efficiency over a range of pH 2 to 7. The percent fluoride removal increased as pH increased from 2 to ≈ 5 and thereafter decreases steadily until pH 10–12. The optimal pH with a fluoride removal of about 48% was observed at pH of 5. At lower pHs, the clays’ surface is positively charged and hence fluoride is electrostatically attracted to the clay surface, which explains higher fluoride sorption between pH 2–5. While at higher pH the sorption of fluoride reduces as the surface becomes negatively charged and therefore a repulsion between the surface and fluoride anions. The chemistry of fluoride removal at different pH could also be explained by relating it to the point of zero charge $\left({\mathrm{pH}}_{\mathrm{pzc}}\right)$ of the adsorbent. ${\mathrm{pH}}_{\mathrm{pzc}}$ refers to the pH at which the adsorbent has zero net charges on the surface. Above the ${\mathrm{pH}}_{\mathrm{pzc}}$ the clay adsorbent is negatively charged and below the ${\mathrm{pH}}_{\mathrm{pzc}}$ the clay adsorbent is positively charged. Higher adsorption at lower pH range of 2–5 lead to the assumption that chemisorption predominates while lower adsorption at higher pH range of 7–12 is suggestive of physisorption.

#### d) Effect of Fluoride Ion Concentration

There is a sharp decrease in percent fluoride removal (from 32 to 18) as the initial fluoride concentration increases from 1–5 mg/L and thereafter slowly decreases (from 18 to 7) with further increase in the initial fluoride concentration from 5–95 mg/L (Figure 6d). The decrease in fluoride adsorption was due to more fluoride ions in solution at higher fluoride concentrations, competing for fewer positive/binding sites on the clay surfaces. The increase in fluoride adsorption at low initial fluoride concentrations was mainly due to more readily available positive/binding sites on the clays surfaces and lower fluoride ions in solution. Hence an initial fluoride concentration of 3.2 mg/L was taken as the optimum concentration for subsequent experiments.

#### e) Effect of co-Existing Anions on the Defluoridation Process

**Figure 7:**
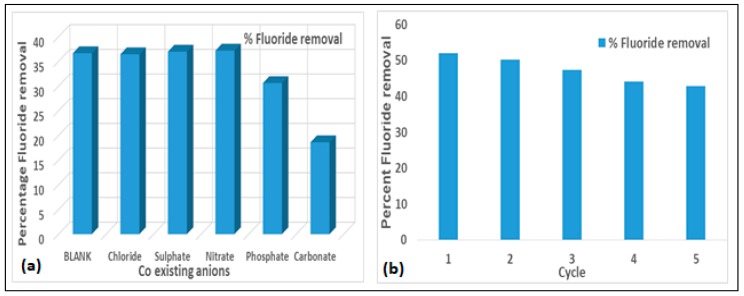
(**a**) Effects of co-existing anions on fluoride removal by the activated clay (at 10 mg/L fluoride, 2.0 g/100 mL adsorbent dosage and 30 min contact time at 250 rpm) and (**b**) Percent fluoride removal by activated clay in successive cycles (at 5.44 mg/L fluoride, pH 6.0 and 60 min contact time at 250 rpm).

Groundwater generally may contain other types of anions, in addition to fluoride ions, which may interfere with the defluoridation process. Figure 7a shows the results of the effect of some co-existing anions on the adsorption of F^−^ by activated clay. The adsorption of F^−^ onto the activated clay is slightly influenced by Cl^−^, as there is little decrease in percent fluoride removal from the blank while SO_4_^2−^ and CO_3_^2−^ gave some slight increase in percent fluoride removal. However, PO_4_^2−^ and CO_3_^2−^ tends to be competing with the F^−^ in the defluoridation process as the percent fluoride removed was reduced from 36.65 to 30.61 and 36.65 to 17.5 respectively. Generally, negatively charged ions are naturally attracted to positively charged ions. The extent of attraction is dependent on the magnitude of charge and size of ion. Usually higher (multivalent) charged anions are more strongly attracted to cations for bonding than the univalent anions. This probably explains why PO_4_^2−^ and CO_3_^2−^ competed more with F^−^ for adsorption than other anions. The order of F^−^ removal in the presence of other co-existing anions is as follows: CO_3_^2−^ > PO_4_^2−^ > Cl^−^ > SO_4_^2−^ > NO_3_^−^.

### 3.5. Regeneration of the Activated Clay Adsorbent

Evaluation of the regeneration of activated clay was performed at an initial natural fluoride concentration of 5.44 mg/L, contact time of 60 min at pH 6.0 in five successive adsorption and desorption cycles The results are presented in Figure 7b. The percent fluoride removal after the first cycle was observed to decrease slightly after each cycle from 52 to 42. The same trend was reported in Mudzielwana et al. [23], Jia et al. [25], and Zhang et al. [26]. The activated clay was observed to be inadequately regenerated, hence further studies with other regenerants are recommended.

### 3.6. Adsorption Mechanisms of Fluoride Ions onto the Activated Clay Soil

The clay soil surface is mainly characterized by Si-OH and Al-OH groups which may be easily modified by changing the pH of the medium. The point of zero charge obtained is about 5–6 where the surface has neutral charge, below 5 the surface is positively charged and above 5 it is negatively charged. Figure 6c shows that fluoride ions adsorption onto the activated clay surface was higher at lower pH up to around 5 and decreases as the pH increases further, which may have been as a result of increase in OH^−^ which competes with fluoride ions for adsorption sites. During fluoride adsorption there was ion exchange between OH^−^ in the surface and fluoride ions in solution, leading to the formation of new bonds such as AlF and SiF. Different mechanisms have been proposed at various pHs. At pH below 5, the surface of the clay is positively charged (Equation (3)), hence fluoride ions are electrostatically adsorbed to the surface of the clay (Equation (4)). Moreover low pH leads to surface protonisation of OH groups to –OH^2+^ which facilitates the ligand exchange mechanism due to stronger attractive forces between fluoride and the adsorbent surface and the presence of more hydroxylated sites for exchange of fluoride at higher pH. At moderate pH (Equation (5)) and pH above point of zero charge (Equation (6)), at which the clays surface is negatively charged, fluoride adsorption both occurs via ion exchange:

≡AC(OH)_2_ + H^+^ ↔ ≡ACOH^3+^ (3)

≡ACOH + H_3_O^+^ + F^−^ ↔ ≡ACOH^2+^ + F^−^ + H_2_O (4)

≡ACOH + F^−^ ↔ ACF + OH^−^ (5)

≡AC(OH)_2_^−^ + 2F^−^ ↔ ACF_2_ + 2OH^−^ (6)

where ≡AC represents aluminium and silicon in the activated clays’ (AC) surface.

### 3.7. Antimicrobial Experiments Using Well Diffusion Assay Method

Antibacterial activities of the mechanochemically activated clay samples were determined by using the well assay method.

**Figure 8:**
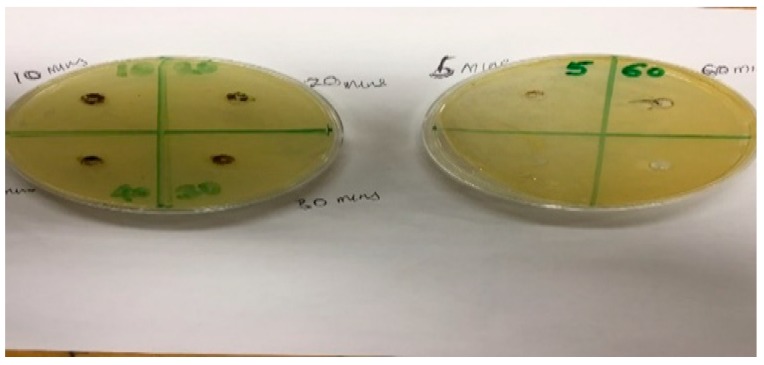
Pictorial view of the plates showing mixtures of *E*. *coli* strains and activated clay samples in the six bored holes.

Figure 8 shows pictorial view of the plates and the six bored holes containing the mixtures of the activated clays and the *E. coli* strains that are being investigated for antibacterial activities via zone of inhibition measurements. The six clay samples were activated at varying contact times of between 5 and 60 min. From Figure 8, it can be observed that all the six samples within the bored holes do not inhibit the bacterial strains and hence they have no zone of inhibition, thereby indicating that they do not have any potent activities against the *E. coli* strains used in this study.

### 3.8. Adsorption Isotherms

Langmuir [27] and Freundlich [28] adsorption isotherms models are equilibrium test used to provide a general idea of adsorbents effectiveness in defluoridation. The adsorption capacity of the activated clay and the nature of the adsorbent surface were determined by finding out if the sorption data fits into Langmuir or Freundlich isotherms. The fluoride ion distribution between the solid and liquid phase is a measure of the position of equilibrium in the adsorption processes which are expressed by Langmuir and Freundlich isotherm models.

**Figure 9:**
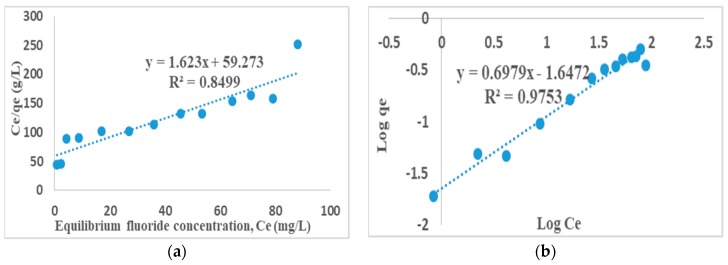
(**a**) Langmuir, and (**b**) Freundlich adsorption isotherms. (10 mg/L fluoride concentrations, 2 g/100 mL adsorbent dosage and 60 min contact time at 250 rpm. Fluoride concentration was varied from 1 to 100 mg/L).

Langmuir adsorption isotherm models the monolayer coverage of the adsorption surfaces and assumes that adsorption takes place on a structurally homogenous surface of the adsorbent. The linear form of the Langmuir model is given as [27]:

(7) $$\frac{1}{q_{e}}=\frac{1}{q_{max}bC_{e}}+\frac{1}{q_{max}}$$

where $C_{e}$ is the concentration of fluoride at equilibrium. $q_{e}$ is the adsorption capacity, $q_{max}$ is the monolayer capacity of the adsorbent and *b* is the Langmuir adsorption constant. The values of Langmuir parameters, $q_{max}$ and *b* was calculated from the slope and intercept of the linear plots of $\frac{1}{q_{e}}$ versus $\frac{1}{C_{e}}$, with regression coefficient (*R*^2^) in Figure 9a.

The essential characteristics of the Langmuir isotherm is denoted by the dimensionless constant called equilibrium parameter, $R_{L}$, which is defined by [29].

(8) $$R_{L}=\frac{1}{1+bC_{0}}$$

where *b* is the Langmuir constant and $C_{0}$ is the initial fluoride concentration (mg/L). The value of $R_{L}<1$ represents favorable adsorption, $R_{L}>1$ unfavorable adsorption, $R_{L}=1$ linear and $R_{L}=0$ indicates irreversible adsorption [30]. 

The plot of *C_e_*/*q_e_* values against *C_e_* for the sorption data at 298 K gave straight line with high correlation coefficients of *R*^2^ = 0.85 (Figure 9a); an indication of a favorable adsorption, possibly a monolayer adsorption of fluoride on the smooth surface of the adsorbent.

Freundlich equation is derived to model the multilayer adsorption on heterogenous surfaces. The linearized form of Freundlich model is given as [28]:

(9) $$\log\;q_{e}=\log k_{F}+\frac{1}{n\log C_{e}}$$

where *C_e_* is the equilibrium concentration, *q_e_* is equilibrium adsorption capacity and $\frac{1}{n}$ are Freundlich constants related to minimum adsorption capacity and adsorption intensity respectively. The values of *k_F_* and $\frac{1}{n}$ are obtained from the slope and intercept of the linear Freundlich plot of log *q_e_* versus log *C_e_* [31].

The plot of log *q_e_* against log *C_e_* for the sorption data at 298 K gave straight line with high correlation coefficients (*R*^2^) (Figure 9b). This is also an indication of favorable adsorption. The Freundlich correlation coefficient (*R*^2^ = 0.98) is however higher than those of Langmuir which is (*R*^2^ = 0.85). Therefore Freundlich isotherm gave a better fit to the sorption data, thereby confirming heterogeneous (multilayer) adsorption process.

### 3.9. Adsorption Kinetic Modelling

Adsorption kinetics studies enables us to experimentally determine the rate of chemical reaction and its dependence on concentration and temperature. The kinetics and mechanism of fluoride sorption onto the activated clays’ surface was investigated in order to understand the mechanism and the rate determining step of the adsorption process by using pseudo-first-order and pseudo-second-order kinetic models.

Pseudo-first-order kinetic model equation is given as Lagergren equation [32]:

(10) $$\log\left(q_{e}-q_{t}\right)=\log\;q_{e}-\frac{k}{2.303}t$$

where $q_{t}$ is fluoride amount on the clays’ surface at time t (mg/g) and k is the equilibrium rate constant of pseudo-first-order sorption (min^−1^).

The straight-line plot of $\log\left(q_{e}-q_{t}\right)$ against t will give the value of the rate constants (k). Linear plot of $\log\left(q_{e}-q_{t}\right)$ against *t* give straight line which shows the applicability of Lagergren equation [32].

The most widely linear form of pseudo-second-order model was used and the equation is given as [33,34]:

(11) $$\frac{t}{q_{t}}=\frac{1}{h}+\frac{t}{q_{e}}$$

where $q_{t}=q_{e}^{2}kt/\left(1+q_{e}kt\right)$), fluoride amount on the adsorbents’ surface at any time, *t* (mg/g), *k* is the pseudo-second order rate constant (g/mgmin), *q_e_* is the amount of fluoride ion sorbed at equilibrium (mg/g) and the initial sorption rate, $h=kq_{e}^{2}$ (mg/gmin). The value of *q_e_* = (1/slope), *k* = (slope^2^/intercept) and *h* = (1/intercept) of the pseudo-second-order equation can be found out experimentally by plotting *t*/*q_t_* against *t*.

**Figure 10:**
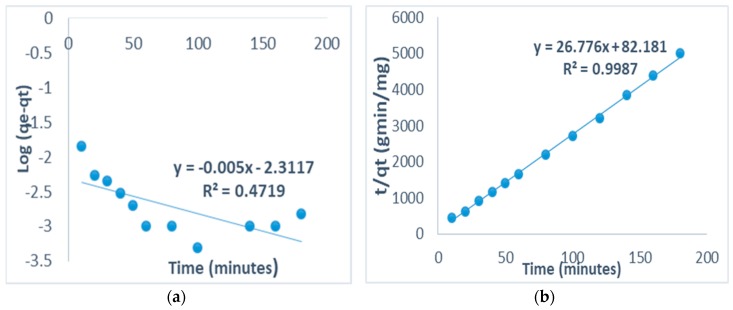
(**a**) Pseudo-first order and (**b**) Pseudo-second order model of fluoride adsorption onto the activated clay.

The fitness of the pseudo-second-order model (Equation (11)) on the sorption of fluoride on the clays’ surface was investigated. The plot of t versus *t*/*q_t_* gives a straight line with higher correlation coefficient, *R*^2^ = 0.99, which is much higher than that observed with pseudo-first-order model (*R*^2^ = 0.4719) indicating that the pseudo-second-order model is more applicable and viable (Figure 10a,b).

From the plot, the values of the rate constant, *k* (min^−1^)) for second order model, *k* = 8.7240 (min^−1^) is higher than first order rate which is 0.0115 (min^−1^) hence indicating chemisorption.

### 3.10. Intra-Particle Diffusion Model

The solute transfer in a solid-liquid sorption process is usually characterized either by particle or pore diffusion control. The intra-particle diffusion controlled sorption process equation [35,36] is given as:

(12) $$In\;\left(1-\frac{Ct}{Ce}\right)=-k_{p}t$$

where *k_p_* is the particle rate constant (min^−1^). The particle rate constant value is obtained by plotting the slope of ln (1 − *Ct*/*Ce*) against *t*.

The simple intra-particle diffusion model was proposed by Weber and Morris [37,38,39,40] and the equation is given as:

*q_t_* = *k_i_ t*^1/2^ (13)

where *k_i_* is the intra-particle rate constant (mg/g min^0.5^).

The correlation coefficient value, *R*^2^ = 0.7801, indicates the sorption process is controlled greatly by the particle diffusion while *R*^2^ = 0.2268 indicates that the sorption process is controlled by pore diffusion models.

The slope of the plot of qt against t^½^ gives the intra-particle rate constant value. The plot of *qt* versus t^0.5^ gives an initial curve followed by a straight line which is suggestive of two types of mechanisms involved in the adsorption process. The initial curve represents the boundary layer effect while the linear part corresponds to intra-particle diffusion.

**Figure 11:**
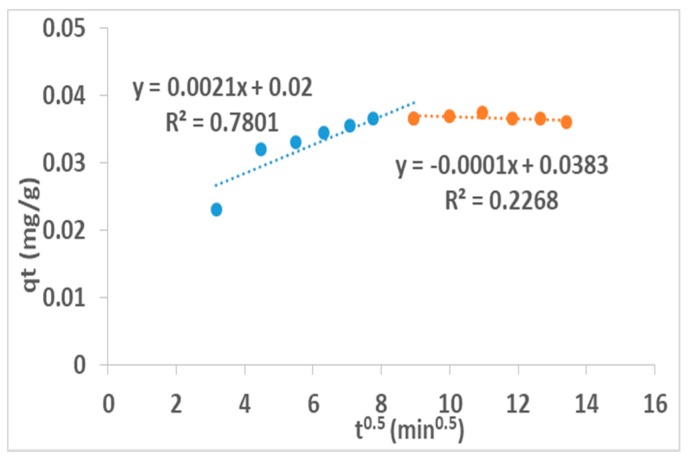
Intra-particle diffusion model of fluoride adsorption on the activated clay.

Both particle (Equation (12)) and pore (Equation (13)) diffusion models have been applied and the values of *k_p_* and *k_i_* shows that *k_p_* = 1.0 × 10^−4^ min^−1^ while *k_i_* = 2.1 × 10^−3^ mg/min^1^^/2^. Higher correlation coefficient, *R*^2^ = 0.7801 indicates the possibility of sorption process being controlled greatly by the particle diffusion while pore diffusion models with (*R*^2^ = 0.2268) is minimal in the sorption process (Figure 11). The constant values for adsorption isotherms and kinetics model is given in Table 6.

### 3.11. Bench Scale Pilot Testing of (Siloam) Groundwater

The efficiency of activated clay in fluoride removal from water was tested on a bench scale pilot groundwater from Siloam village containing 5.44 mg/L initial fluoride concentration at optimum initial pH 6.0 and at a natural pH of about 8.5 respectively. Anion and cation concentrations before and after treatment are presented in Table 7. There was no major change in the concentrations of Cl, Br, and SO_4_^2−^ before and after treatment, indicating that these co-existing anions do not compete in the defluoridation process, while there was reduction in the concentration of PO_4_^2−^ from 4.84 to 2.19 mg/L, representing a 40.4% removal and suggesting that PO_4_^2−^ competed with F^−^ in the defluoridation process. NO_3_^2−^ was not detected (ND). The percent F^−^ removal was 21. This is lower than the percent F^−^ removal of 32 in the simulated fluoride water. This may be due to the presence of more OH groups at this higher pH which competed with F^−^. Furthermore, the F^−^ concentration in the raw pilot water before and after treatments at different pH of 6.0 and 8.5 are still much higher than the permissible WHO limits. This suggest that the adsorbent was not effective but could be subjected to further modification thereby increasing its surface area and adsorptive capacity. The concentrations of all the cations were slightly higher after treatments. This was due to some cationic exchanges into the media, but are still within WHO permissible limits. Hence, the activated clay is safe and therefore recommended for remediation of fluoride in drinking water.

## 4. Conclusions

The maximum adsorption capacity of activated clay in simulated F^−^ water was found to be 1.87 mg/g with 32% F^−^ removal. Fluoride uptake reduced in the presence of Cl^−^, PO_4_^3−^ and CO_3_^2−^ while it increased in the presence of SO_4_^2−^ and NO_3_^−^. None of the activated clays showed any activities against the tested *E. coli* strains, hence suggesting limited antibacterial properties of their surfaces. The adsorption models for Langmuir and Freundlich isotherms showed a favorable adsorption process. The Freundlich correlation coefficient (*R*^2^ = 0.98) is higher than the Langmuir one (*R*^2^ = 0.85), therefore, the Freundlich isotherm gave a better fit, thereby confirming a heterogeneous (multilayer) adsorption process. The kinetics correlation coefficient (*R*^2^ = 0.99) is much higher for pseudo-second order than pseudo-first order value (*R*^2^ = 0.47), hence F^−^ adsorption was fitted well to a pseudo-second order model suggesting that the adsorption occurs via a chemisorption pathway. The F^−^ sorption was found to be intra-particle diffusion controlled. The high correlation coefficient indicates the possibility of the sorption process being controlled greatly by the particle diffusion, while in pore diffusion models it is minimal. The defluoridation of Siloam groundwater at initial pH 6.0 and natural pH of about 8 showed a 21% F^−^ removal which is much lower than the 32% observed in the simulated F^−^ water, which is probably due to some anions competing with F^−^ during defluoridation in the Siloam groundwater. Concentrations of all the cations were slightly higher after treatment due to some cationic exchanges into the media, but are still within WHO permissible limits. All the anions except F^−^ were within WHO permissible limits after treatment, suggesting the adsorbent’s inefficiency in reducing the F^−^ to permissible limits. Hence, the activated clay is safe and recommended for fluoride remediation in drinking water. The adsorption efficiency and antibacterial potency of the activated clay for defluoridation and pathogen removal could be improved by further modification of its surfaces and introduction of suitable antibacterial metal oxides.

## Figures and Tables

**Table 1:** Fluoride levels in clay soil samples activated at different contact times.

Samples	Mechanochemically Activated Time (min)	Fluoride Level (mg/L)	Fluoride Level (mg/g)
A	5	0.0401	2.0 × 10^−3^
B	10	0.0610	3.05 × 10^−3^
C	20	0.0605	3.02 × 10^−3^
D	30	0.0638	3.19 × 10^−3^
E	40	0.0638	3.19 × 10^−3^
F	60	0.0703	3.51 × 10^−3^

**Table 2:** The surface area, pore volume and pore size of the activated clay soils.

Samples	Mechanochemically Treatment Time (min)	BET Surface Area (m^2^/g)	Surface Area Single Point (m^2^/g)	Pore Volume (cm^3^/g)	Pore Size (nm)
A	5	16.2717	15.5624	10.038973	5.93219
B	10	15.6702	15.2082	10.048930	11.57293
C	20	16.0833	15.6099	10.052368	12.05213
D	30	17.1991	16.6604	10.071315	14.92835
E	40	16.5091	15.9606	10.070300	15.15535
F	60	13.2293	12.5760	10.054879	14.67643

**Table 3:** Cation exchange capacity of activated clay in milliequivalents/100 g with exchangeable chemical species.

Parameter	pH	Mg^2+^	Ca^2+^	Na^+^	K^+^	Total CEC (milliequivalents/100 g)
CEC	5.4	34.6	17.7	8.1	14.3	74.5
	7.4	38.1	19.5	9.0	15.4	82.1

**Table 4:** Major and minor elemental composition (%).

SiO_2_	TiO_2_	Al_2_O_3_	Fe_2_O_3_	MnO	MgO	CaO	Na_2_O	K_2_O	P_2_O_5_
52.48	0.627	14.62	6.64	0.125	2.985	1.53	0.707	1.24	0.034

**Table 5:** Percent fluoride removal of the activated clays at different treatment times and their adsorption capacities.

Samples	Mechanochemically Activated (min)	Initial Fluoride Concentration *C*_0_ (mg/L)	Equilibrium Fluoride Concentration *C_e_* (mg/L)	Difference between Initial and Final Fluoride Conc (mg/L)	% of Fluoride Removed	Adsorption Capacity, q (mg/g)
A	5	8.39	6.45	1.94	23.12	0.97
B	10	8.39	6.51	1.88	22.41	0.94
C	20	8.39	6.43	1.96	23.36	0.98
D	30	8.39	6.00	2.39	28.49	1.195
E	40	8.39	6.22	2.17	25.86	1.085
F	60	8.39	6.56	1.83	21.81	0.915

**Table 6:** Constant values for adsorption isotherms and adsorption kinetics model.

**Langmuir Isotherm**			**Freundlich Isotherm**				
*Q*_m_ (mg/g)	*b* (L/mg)	*R* ^2^	*K_F_* (mg/g)	1/*n*	*R* ^2^		
0.62	0.0273	0.85	44.38	0.69	0.98		
**Pseudo First Order**			**Pseudo Second Order**		**Intra-Particle Diffusion**		
*q_e_*_(exp)_ (mg/g)	*K_ad_* (min^−1^)	*R* ^2^	*K_2ad_* (g/mg min)	*q_e_*_(cal)_ (mg/g)	*R* ^2^	*K_i_*	*K_p_*
−6.93	1.15 × 10^−2^	0.47	8.72	3.73 × 10^−2^	0.99	2.1 × 10^−3^	1 × 10^−4^

**Table 7:** Physicochemical properties of raw and treated pilot fluoride rich groundwater.

Parameters	Ions Concentrations in Raw Pilot Water before Treatment (mg/L)	Ions Concentrations in Treated Pilot Water (mg/L)	Ions Concentrations in Treated Pilot Water (mg/L)	WHO Permissible Limits (mg/L)
pH	8.0	6.0	8.5	6.5–8.5
F^−^	5.338	4.174	4.271	1–1.5
Cl^−^	5.078	34.150	34.255	250
Br^−^	0.409	0.388	0.408	200
SO_4_^2−^	12.212	12.213	12.213	200–400
PO_4_^3−^	4.84	2.198	2.019	20–50
NO_3_^−^	ND	ND	ND	50–100
Na+	65.44	102.30	81.50	200–250
K^+^	19.4	68.50	66.30	200–250
Ca^2+^	2.75	3.15	6.80	75
Mg^2+^	21.25	22.55	51.50	50
